# The Story of the Insane from Year to Year

**Published:** 1893-02-25

**Authors:** 


### THE STORY OF THE INSANE FROM YEAR TO YEAR.

(Fifth Series.)

### OXFORD COUNTY ASYLUM AT LITTLEMORE.

From the annual report for 1891 we find that the number of patients increased during the year from 483 to 516. The admissions reached 115, the recoveries 22, and the deaths 41. The percentage of recoveries stands at 26, and the deaths at 8. Hereditary tendency was the cause of the insanity in 28 of the cases admitted, and 6 were driven mad by drink. "Disease of the lungs" is the assigned cause of death in 16 instances, and post-mortem examinations were made in every case of death except one. We have elsewhere referred to this wholesale making of post-mortem examinations in county asylums, and we need not recapitulate our remarks here further than to say that it is very unlikely all these were necessary. The visitors' report is a somewhat long one, and we learn one useful thing from it, namely, that the "Government auditor has given his decision, based on authority, that all rates and taxes in respect of the buildings, grounds, and premises are chargeable to the repairs account." It is satisfactory to have this decision made public, as much doubt has hitherto prevailed on the subject. The weekly rate is 8s. 2d.

### WONFORD HOUSE, EXETER.

This Hospital for the Insane contained 124 patients on January 1st, and it had 125 in residence at the close of the year. Twenty-five patients were admitted; 13 were discharged recovered, and 7 died. The percentage of recoveries stands at 61·9, and the deaths at 5·5. Influenza turns up as a cause in 3 of the admissions, and hereditary tendency in 5; while we do not notice drink given as the cause in a single case, nor does consumption appear as a cause of death. Dr. Deas finds that medical men are as unwilling as ever to sign certificates of insanity, as the protection thought to be obtained by the new Act is more or less illusory. Even in a simple point like this the wretched Act would seem to have failed; and, says Dr. Deas, "the cumbrous machinery of the Act only frightens people, and often makes them hesitate to act when action is necessary." It is very satisfactory to note that nearly one-half of the patients admitted were received at a reduced rate of maintenance, the average being 25s. It would be very desirable if this and all similar hospitals were to publish details of these payments similar to those given by the Warneford Hospital at Oxford, and also show the average cost per head per week like county asylums. Here it was 35s. 6d. Influenza appeared in November. It attacked many of the staff and eight of the patients. Dr. Deas has some very good remarks on restraint. He tells us he used it in three cases during the year, and that in one of these he believes the patient's life and reason were saved by it. Dr. Deas says there is a tendency in some quarters to blame anyone who honestly uses "restraint" as treatment, and possibly there is such a tendency; but it is very silly, and Dr. Deas can well afford to put it down to ignorance.

### NOTTINGHAM BOROUGH ASYLUM, MAPPERLY HILL.

The twelfth annual report tells us that 544 patients were in residence at the beginning of the year, and that this number had increased to 550 at the close of the year. The admissions were 139, the recoveries 53, and the deaths 56. The recoveries work out at 41 per cent. on the admissions, and the deaths at 10·2 per cent. on the average number resident. Taking the whole twelve years during which the asylum has been in existence, the average recovery rate stands at 42·3 per cent., and the death rate at 11·2. Turning to the table showing the assigned causes of insanity in those admitted during the year, we find that drink is given in 30 cases, hereditary tendency in 20, previous attacks in 22, influenza in 6, and in 21 the cause was not ascertained. Six of the deaths were caused by consumption. We notice that 109 of the patients are chargeable to the county of London, the charge for each of these being 13s. a week. As the average cost per head in the English County Asylums is about 8s. 6d. a week, the County of London is losing annually a sum of something like £1,250 a year by this arrangement, and it is said that even larger sums are being lost. The visitors' report is a model of conciseness. We did not notice among the tables any statement of the average cost of maintenance; but we learn from the Commissioners' report that it is 10s. 6d. There is a balance of £475 in favour of the farm, and the poor Nottinghamshire farmer, who cannot make ends meet, will doubtless ask, "Pray, how do they manage to do it?"

### CUMBERLAND AND WESTMORELAND ASYLUM, CARLISLE.

An increase of only four patients during the year has to be recorded at this asylum. There were 149 admissions, 79 recoveries, and 53 deaths. At the close of the year there were 575 patients in the asylum. The recoveries stand at 53 per cent. of the admissions, and the deaths at 9 2 on the average number resident. Hereditary influence accounts for 42 of the admissions, drink for 25; previous attacks for 29 and in 56 the cause of the insanity was not ascertained. Only four of the deaths were due to consumption. When speaking of the high recovery rate, Dr. Campbell says that "one year's good recovery rate means nothing; but a high average over a long course of years surely does." No doubt; but what does it mean? That's the rub. And again, Dr. Campbell says there is in some asylums "a tendency to promulgate the idea that the sanguine mind of a superintendent in estimating the mental condition of a patient on leaving influences the recovery rate." Perhaps the recovery rate is so influenced, as it is likely enough that the standard of sanity whereby insanity is judged, may be different among medical superintendents, and what one would call "recovered" another would put down as "relieved." At any rate, if we put on one side cases relieved and cases readmitted, we should probably find that any existing difference in the recovery rate would be accounted for by the nature of the admissions. For instance, on page 22 of this report Dr. Campbell publishes a table, in which he places the number of probably curable cases in his asylum at 50, and we doubt whether any other asylum in England has such a high proportion of curable cases in its wards. The committee say they are perfectly satisfied with the condition, management, and superintendence of the asylum." The weekly cost per head is nearly 8s. 11d.

### NORTHAMPTON COUNTY ASYLUM, BERRY WOOD.

On the first day of 1891 there were 753 patients in this asylum, and by the end of the year the numbers had risen to 851. The admissions reached 280, the recoveries 67, and the deaths 81. The recoveries stand at 39·4 per cent. on the admissions, and the deaths at 9·9 per cent. on the average number resident. Hereditary tendency is said to have caused the insanity in 70 of the admissions and drink in 20, and Mr. Greene says that "putting the hereditary cases and the drink cases together, it is seen that over 50 per cent. of the admissions of the year 1891 had no right to exist at all." And he adds that such facts when brought to light ought to sound a warning note, and they are certainly a terrible satire on the boasted civilization of the nineteenth century." There were 11 deaths from consumption and 3 from enteritis, the latter being presumably that form of dysentery which has lately prevailed in many of our county asylums. Mr. Greene's views as to its cause seem to agree with those of Dr. Sheldon, of Macclesfield, quoted in a previous notice. The Lunacy Act is subjected to a long and unfavourable criticism, ending as follows: "The Medical Superintendent has pointed out some of the anomalies of the Act in the hope that the visitors may use their influence to obtain the repeal of its objectionable clauses." We heartily join in this hope. There is a balance of £218 in favour of the farm, and this has been obtained after estimating rent o land, interest on capital, and patients' labour; but the pric of produce supplied by the farm to the asylum are not give . The weekly rate is 7s. 6d. per head.

